# Catalytic Determinants of Alkene Production by the Cytochrome P450 Peroxygenase OleT_JE_*

**DOI:** 10.1074/jbc.M116.762336

**Published:** 2017-01-04

**Authors:** Sarah Matthews, James D. Belcher, Kang Lan Tee, Hazel M. Girvan, Kirsty J. McLean, Stephen E. J. Rigby, Colin W. Levy, David Leys, David A. Parker, Richard T. Blankley, Andrew W. Munro

**Affiliations:** From the ‡ Manchester Institute of Biotechnology, School of Chemistry, University of Manchester, Manchester M1 7DN, United Kingdom,; the § Westhollow Technology Center, Houston, Texas 77028-3101, and; ¶ Agilent Technologies UK Ltd., Cheshire SK8 3GR, United Kingdom

**Keywords:** crystal structure, cytochrome P450, decarboxylase, enzyme mechanism, fatty acid oxidation, CYP152L1, EPR spectroscopy, alkene, fatty acid hydroxylation, product analysis

## Abstract

The *Jeotgalicoccus* sp. peroxygenase cytochrome P450 OleT_JE_ (CYP152L1) is a hydrogen peroxide-driven oxidase that catalyzes oxidative decarboxylation of fatty acids, producing terminal alkenes with applications as fine chemicals and biofuels. Understanding mechanisms that favor decarboxylation over fatty acid hydroxylation in OleT_JE_ could enable protein engineering to improve catalysis or to introduce decarboxylation activity into P450s with different substrate preferences. In this manuscript, we have focused on OleT_JE_ active site residues Phe^79^, His^85^, and Arg^245^ to interrogate their roles in substrate binding and catalytic activity. His^85^ is a potential proton donor to reactive iron-oxo species during substrate decarboxylation. The H85Q mutant substitutes a glutamine found in several peroxygenases that favor fatty acid hydroxylation. H85Q OleT_JE_ still favors alkene production, suggesting alternative protonation mechanisms. However, the mutant undergoes only minor substrate binding-induced heme iron spin state shift toward high spin by comparison with WT OleT_JE_, indicating the key role of His^85^ in this process. Phe^79^ interacts with His^85^, and Phe^79^ mutants showed diminished affinity for shorter chain (C10–C16) fatty acids and weak substrate-induced high spin conversion. F79A OleT_JE_ is least affected in substrate oxidation, whereas the F79W/Y mutants exhibit lower stability and cysteine thiolate protonation on reduction. Finally, Arg^245^ is crucial for binding the substrate carboxylate, and R245E/L mutations severely compromise activity and heme content, although alkene products are formed from some substrates, including stearic acid (C18:0). The results identify crucial roles for the active site amino acid trio in determining OleT_JE_ catalytic efficiency in alkene production and in regulating protein stability, heme iron coordination, and spin state.

## Introduction

**FIGURE 1.**
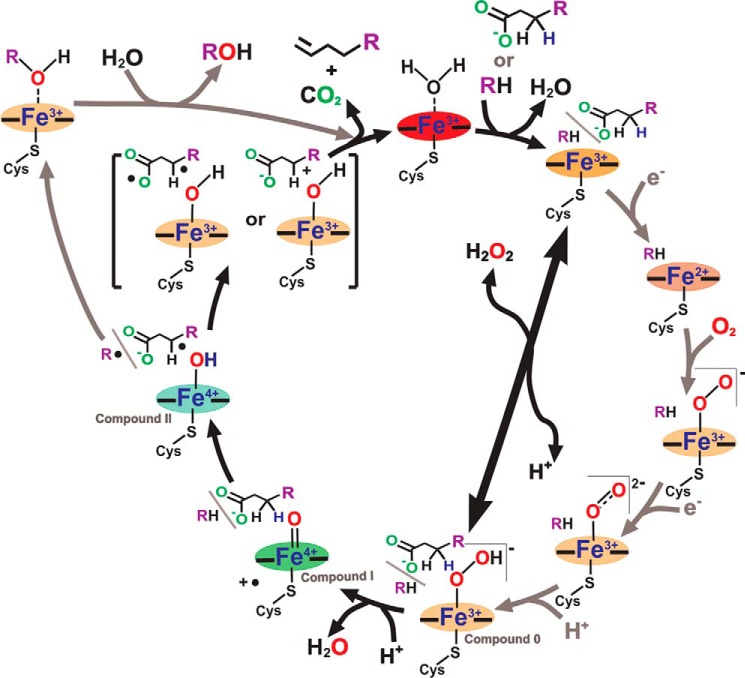
**The OleT_JE_ catalytic cycle.** OleT_JE_ catalyzes the oxidative decarboxylation of fatty acids (major reaction) and can also hydroxylate fatty acids at the α- and β-positions (minor reaction). The canonical P450 catalytic cycle is shown in circular representation, initiating with the binding of fatty acid substrate (shown as RH, and also as a chemical structure) that converts the LS OleT_JE_ P450 to a HS form. Shown in *gray arrows* from this point onwards are reduction (×2) and protonation (×1) steps that follow the canonical path and require one or more redox partners. The *thick black arrow* traversing the cycle illustrates the “peroxide shunt” pathway by which ferric, fatty acid-bound OleT_JE_ can be converted directly to the transient ferric-hydroperoxo (compound 0) form using H_2_O_2_. At this point, the canonical and peroxide shunt pathways converge with a protonation step that results in dehydration of a transient intermediate to form the ferryl-oxo compound I. Compound I deprotonates the substrate, producing a substrate radical and the ferryl-hydroxo compound II. At this stage, the catalytic pathways for substrate hydroxylation and decarboxylation diverge. Hydroxylated fatty acids are formed by the “radical rebound” mechanism (5), as shown in the outer loop to the *left* of the main cycle (*gray arrows*). However, recent studies have indicated that that fatty acid decarboxylation to form terminal alkenes likely involves an unusually stable compound II and that reduction of compound II (or another oxidant) by the substrate radical could form a substrate diradical or carbocation species that decomposes with CO_2_ release to form the terminal alkene product (15, 39). The P450 heme (illustrated by the iron in the relevant oxidation state and equatorial/axial bonds) is *shaded* to approximate the color of the relevant heme intermediate species.

The cytochromes P450 (P450s or CYPs) are a superfamily of heme *b*-containing mono-oxygenase enzymes widespread in nature and spanning all three domains of life: archaea, bacteria, and eukaryota (1). Most P450s catalyze reductive scission of dioxygen bound to their heme iron, relying on the delivery of two electrons acquired from pyridine nucleotide cofactors (NADPH or NADH). In the canonical P450 catalytic cycle, two electrons are donated at discrete points in the cycle via a redox partner protein (2). The first electron is delivered to the substrate-bound, cysteine thiolate-coordinated ferric heme iron, reducing it to the ferrous form, allowing dioxygen to bind. The second electron reduces this ferric-superoxo complex to the ferric-peroxo state. As shown in Fig. 1, two successive protonations of iron-oxo species then occur. Protons are typically relayed from a conserved acid-alcohol amino acid pair (*e.g.* Asp^251^/Thr^252^ in the camphor hydroxylase P450cam, CYP101A1) to form first the ferric-hydroperoxo species (compound 0) and then the highly reactive ferryl-oxo, heme radical cation species compound I (with loss of a water molecule) (3, 4). The P450 compound I abstracts a hydrogen from the substrate to form the ferryl-hydroxo (compound II) form, prior to oxidizing the substrate to complete the catalytic process (5).

Although most P450s operate in this way, several are also known to catalyze substrate oxidation using the “peroxide shunt” mechanism, in which H_2_O_2_ or an organic hydroperoxide (*e.g. meta*-chloroperoxybenzoic acid) convert a substrate-bound resting state of the P450 directly to compound 0 (Fig. 1) (4, 6). As in the canonical cycle, a further protonation of compound 0 leads to formation of compound I and to oxidative catalysis. In many P450s, this reaction is inefficient, and oxidative damage to the protein and/or heme occurs alongside any productive turnover (7). However, certain P450s have evolved to use the peroxide shunt efficiently for substrate oxidation. The best characterized P450s are in the CYP152 family of peroxygenases. These enzymes may have evolved in ancient prokaryotes at a time when the environment was devoid of oxygen, but relatively rich in hydrogen peroxide and peroxygenated organic compounds. Although peroxygenase activity is likely to have evolved in archaea, it has been conserved in P450s from bacteria and eukaryotes, including humans (8).

Among the best characterized of the CYP152 family peroxygenases is the industrially relevant OleT_JE_ (CYP152L1), first isolated from *Jeotgalicoccus* sp. ATCC 8456 in 2011 by Schirmer and co-workers (9). OleT_JE_ acts primarily as a H_2_O_2_-dependent fatty acid decarboxylase to form terminal alkenes but also hydroxylates a proportion of these substrates to generate α- and β-hydroxylated fatty acids. Lipid substrates with chain lengths ∼C12-C20 were shown to be good substrates (9, 10). Later studies showed that the P450cam redox partner system (NADH-putidaredoxin reductase and putidaredoxin), along with a NADH recycling system) could also be used effectively with OleT_JE_ to decarboxylate alkanoic acids as short as C4 (11). OleT_JE_ also functions as a typical P450 mono-oxygenase when fused to the phthalate dioxygenase reductase-like domain of CYP116B2 (RhFRED) from *Rhodococcus* sp. NCIMB9784 (12) or by utilizing the flavodoxin/flavodoxin reductase system of *Escherichia coli in vivo* (12, 13). OleT_JE_ is also active when used in conjunction with a light-driven, *in situ* H_2_O_2_-generating system (14).

Transient formation of P450 compound I in OleT_JE_ was reported by Makris and co-workers (15) on the addition of H_2_O_2_ to OleT_JE_ in complex with perdeuterated arachidic acid, and identified by the appearance of a characteristic 370-nm Soret maximum and an additional absorption band at 690 nm. These spectral data are similar to those reported by Rittle and Green (4) in studies with *Sulfolobus acidocaldarius* CYP119A1. The transient capture of compound I in the perdeuterated substrate-bound form of OleT_JE_ likely results from a ^2^H-kinetic isotope effect on compound I decay. Compound I formation is considered to initiate fatty acid decarboxylation by abstracting a substrate hydrogen atom (15).

As an alkene-producing enzyme, OleT_JE_ has attracted interest for industrial applications, including in the production of biofuels and petrochemicals (16). *In vivo* production of alkenes was reported in *E. coli* strains transformed with OleT_JE_ (12). Yan *et al.* (17) also developed systems in which the tandem activities of a lipase and OleT_JE_ were used to produce 1-alkenes from low cost triacylglycerol feedstocks.

Other prominent members of the CYP152 peroxygenase family include *Bacillus subtilis* P450 BS_β_ (CYP152A1, 41% sequence identity to OleT_JE_) and *Sphingomonas paucimobilis* P450 SP_α_ (CYP152B1, 37% sequence identity to OleT_JE_) (10). Both enzymes are primarily fatty acid hydroxylases, with P450 SP_α_ hydroxylating 100% at the C_α_ position, and P450 BS_β_ initially reported to hydroxylate at ∼43% in the C_α_ position and ∼57% in the C_β_ position (18). However, studies by Rude *et al.* (9) showed that P450 BS_β_ produced 1-pentadecene at ∼15% of total product alongside α- and β-hydroxylated palmitic acid products. Crystal structure data show that the active sites of OleT_JE_ and P450 BS_β_ are very similar, with the carboxylate group of fatty acid substrates being coordinated by a conserved arginine residue (Arg^245^ in OleT_JE_, Arg^242^ in P450 BS_β_, and Arg^241^ in P450 SP_α_) (10, 18). An R242A mutant of P450 BS_β_ abolished fatty acid hydroxylase activity, whereas an R242K mutation substantially reduced substrate hydroxylation (19).

A key difference in the active sites of OleT_JE_ and P450 BS_β_ is at position 85 (His^85^ in OleT_JE_ and Gln^85^ in P450 BS_β_) (10). In OleT_JE_, the His^85^ imidazole side chain points into the active site toward the heme iron. This orientation sandwiches the imidazole side chain between the heme and Phe^79^. It was postulated that the difference in the nature of the residue at position 85 may underlie the strong decarboxylase activity of OleT_JE_. His^85^ could act as a proton donor to the compound I intermediate, together with its reduction to compound II using an electron from the fatty acid substrate carboxylate group. Homolytic scission of the C-C_α_ bond of the fatty acid, concomitant with abstraction of a hydrogen from C_α_ to form compound II, could then produce the terminal alkene and CO_2_ products (10). Rude *et al.* investigated the role of the residue at position 85 in P450 BS_β_ by creating the Q85H mutant. During *in vitro* turnover experiments with hexadecanoic acid, the P450 BS_β_ Q85H mutation resulted in decreased α-hydroxy-hexadecanoic acid formation and in increased 1-pentadecene and β-hydroxy hexadecanoic acid formation. Despite this, the ratio of decarboxylation to hydroxylation only increased from 0.19 in WT P450 BS_β_ to 0.30 in the P450 BS_β_ Q85H mutant, compared with a ratio of 3.32 in OleT_JE_ (9).

Through its interactions with His^85^ in OleT_JE_, Phe^79^ is also likely to be important for catalytic activity in OleT_JE_. A phenylalanine (Phe^79^) is retained at this position in P450 BS_β_. However, the exclusive fatty acid α-hydroxylase P450 SP_α_ has a leucine at the corresponding position (Leu^79^). In P450 BS_β_, the F79L mutation resulted in increased α-hydroxylation, with levels of β-hydroxylation falling from 58 to 17% of the overall product (20).

**FIGURE 2.**
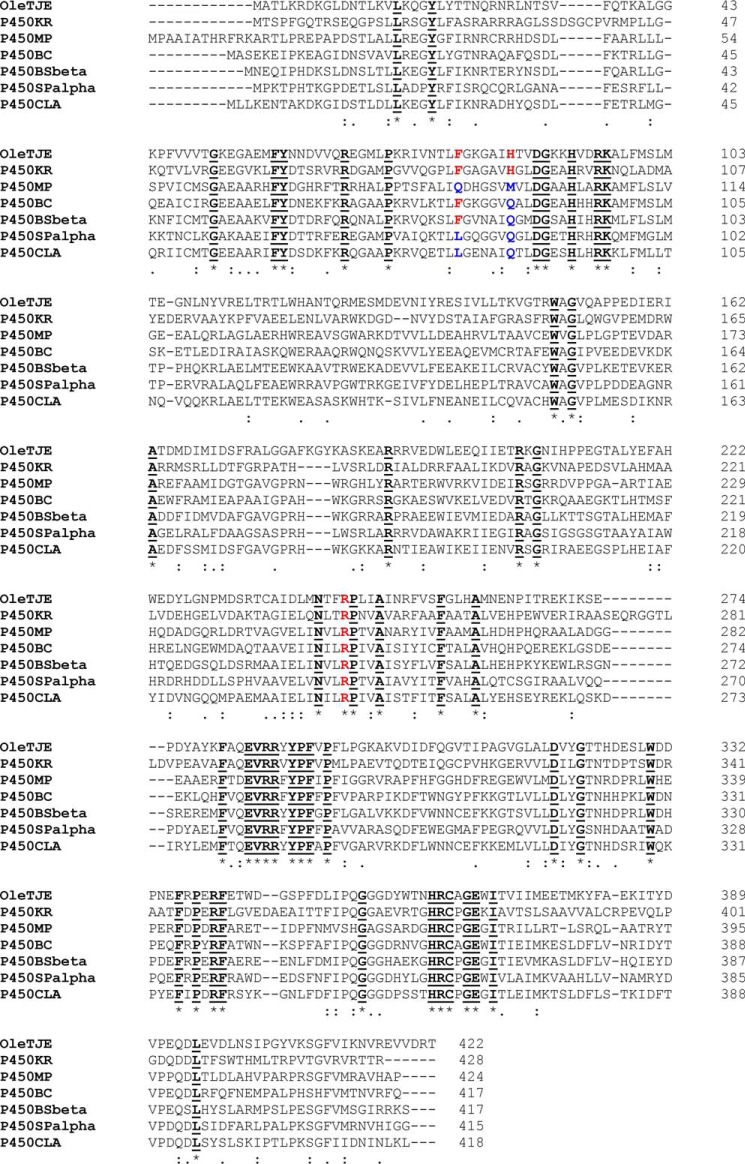
**Alignment of OleT_JE_ with other bacterial peroxygenase P450s.** OleT_JE_ (CYP152L1) is aligned with the peroxygenases P450 KR from *K. rhizophila*, CYP-MP (P450MP) from *M. populi* (30), a peroxygenase from *Bacillus clausii* (P450BC) (9), *B. subtilis* P450 BS_β_ (CYP152A1) (27), *S. paucimobilis* P450 SP_α_ (CYP152B1) (28), and *C. acetobutylicum* P450 CLA (CYP152A2) (33). The OleT_JE_ active site residues Phe^79^, His^85^ and Arg^245^ are in *bold*, *red font*. Non-conserved residues at these positions are shown in *bold*, *blue font*. Arg^245^ and the adjacent Pro^246^ are conserved in all the peroxygenases. Other completely conserved residues are indicated in *bold*, *underlined black font*.

In this study, we explore further the structure and mechanism of OleT_JE_ by mutating the key active site residues His^85^, Phe^79^, and Arg^245^ and by analyzing their effects on the catalytic properties of this biotechnologically important alkene-producing enzyme. The H85Q mutant was characterized to establish whether His^85^ is crucial to OleT_JE_ decarboxylase activity and also because its replacement by Glu^85^ in P450 BS_β_ results mainly in fatty acid hydroxylation (Fig. 2). The importance of Phe^79^ in its interactions with His^85^ and in regulating active site structure and catalytic activity were explored by characterization of F79A, F79W, and F79Y variants. Finally, the fatty acid carboxylate coordinating Arg^245^ was mutated to neutral (R245L) and charge reversal (R245E) variants to assess influence on activity and regioselectivity of substrate oxidation. Mutants were characterized by a combination of kinetic, spectroscopic, structural, and analytical methods to provide new insights into OleT_JE_ structure/function.

## Results

### 

#### 

##### Expression and Purification of OleT_JE_ and OleT_JE_ Mutants

The expression, purification and structural characterization of the WT OleT P450 were reported by Belcher *et al.* (10). The structural data for the substrate-bound and substrate-free forms of OleT_JE_ were used to design mutations to explore the fatty acid carboxylate binding site of the P450. The codon-optimized OleT_JE_ (*CYP152L1*) gene was cloned into pET15b, and site-directed mutagenesis was done to create the H85Q, F79A, F79W, F79Y, R245E, and R245L mutants. All mutants were found to express reasonably well in the *E. coli* C41 (DE3) strain. Typical OleT_JE_ mutant recovery was ∼15 mg/liter for H85Q and F79W, 8 mg/liter for F79Y and R245L, 2 mg/liter for F79A, and 1.5 mg/liter for the R245E OleT_JE_ mutant, compared with ∼20 mg/liter for WT OleT_JE_. The heme precursor δ-aminolevulinic acid (500 μm) was added at the point of induction of OleT_JE_ mutant gene expression with isopropyl 1-thio-β-d-galactopyranoside to aid heme incorporation. However, heme insertion levels were diminished in the R245E and R245L mutants (∼25–35% heme incorporation). The simultaneous addition of hemin (4 μg/ml) at the point of induction increased heme incorporation by ∼5–10% in these mutants.

**FIGURE 3.**
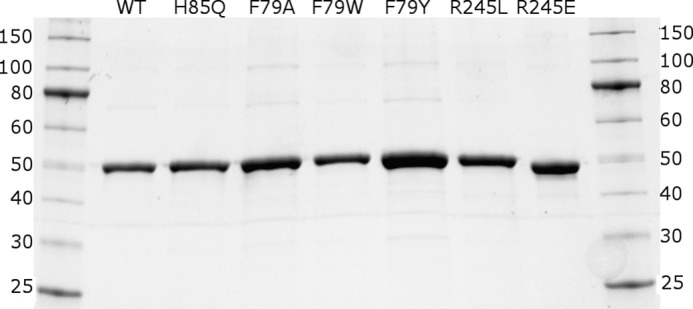
**Purification of WT and active site mutants of OleT_JE_.** The gel image shows the purified forms (from left to right) of WT OleT_JE_ and its active site mutants H85Q, F79A, F79W, F79Y, R245L and R245E. The outside lanes show protein markers with their associated molecular masses (kDa) indicated (NEB Broad Range Protein Ladder).

All OleT_JE_ mutants were purified in the same way, using nickel-iminodiacetic acid (Ni-IDA)^2^ chromatography. Samples for enzymatic/spectroscopic assays were frozen after ensuring a high level of purity by SDS-PAGE. Samples destined for crystallography were treated with TEV protease to cleave the N-terminal His tag and then passed through a Ni-IDA column to separate the untagged form from column-retained tagged OleT_JE_. Gel filtration chromatography was used to further purify untagged OleT_JE_ proteins, and samples were then used directly in crystallographic trials. Fig. 3 shows an SDS-PAGE gel containing samples of purified WT and mutant OleT_JE_ proteins. The F79W, F79Y, R245E, and R245L OleT_JE_ mutants proved less stable than the WT and the H85Q and F79A mutants in that they were prone to aggregation and precipitation on refrigeration or during crystallization trials. However, the H85Q and F79A OleT_JE_ P450s were successfully crystallized for structural analysis (see “Structural Properties of OleT_JE_ Mutant Enzymes” below).

##### UV-visible Absorbance Properties of OleT_JE_ Mutants

When purified from *E. coli*, the WT, ferric OleT_JE_ exhibits a mixed low spin (LS)/high spin (HS) heme iron state that can be converted essentially completely to a LS form by the addition of H_2_O_2_. This likely indicates that fatty acids from *E. coli* are bound and retained by the enzyme during the purification process. The WT OleT_JE_ was extensively dialyzed to remove bound fatty acids. However, this mixed spin character was absent in all OleT_JE_ mutants generated, suggesting that the mutations may compromise affinity for mid-chain to long chain fatty acids through structural perturbation of the P450 active site.

**FIGURE 4.**
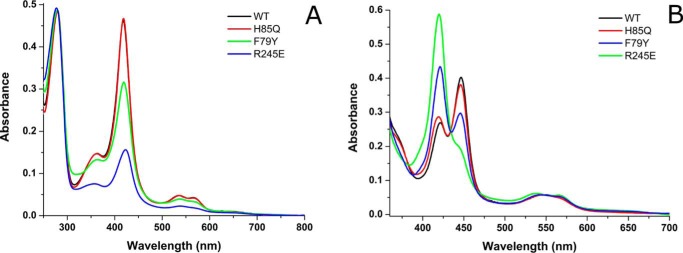
**UV-visible spectroscopy of OleT_JE_ and key active site mutants.** *A* shows typical UV-visible absorption spectra for the ferric, substrate-free forms of WT (*black*), H85Q (*red*), F79Y (*green*), and R245E (*blue*) OleT_JE_. The spectra are nearly identical at the heme Soret and protein (∼280 nm) peaks for the WT and H85Q forms (both at 5 μm) and reflect essentially complete heme incorporation into these proteins. However, the heme/protein (Reinheitszahl, Rz) ratio is much lower in the F79Y and R245E OleT_JE_ mutants, indicating lower heme incorporation in these variants. *B* shows the Fe^II^-CO complexes of the same proteins (corrected to 5 μm by heme content in all cases), showing that the WT and H85Q mutants form predominantly the cysteine thiolate-coordinated P450 form, with a proportion of the thiol-coordinated P420 state. There is a considerably greater proportion of the P420 species in the F79Y mutant, whereas the R245E mutant forms almost completely the P420 form. The P450 species have a Soret maximum at 448 ± 1 nm and the P420 species a Soret maximum at 422 ± 1 nm in all cases.

In WT OleT_JE_, the ferric heme Soret peak is at 418.5 nm, with the α and β bands at ∼567 and 535 nm, respectively. The H85Q mutant has a similar spectrum with a Soret peak at 418.5 nm, whereas the F79A/W/Y mutants have Soret maxima at 419/419/419.5 nm. In the R245E mutant, there is a substantial spectral change, with the Soret peak at ∼423 nm and a major Q-band feature at ∼538 nm. This phenomenon is not observed in the R245L OleT_JE_ (Soret maximum at 417.5 nm, with α/β bands at ∼566/535 nm), indicating that the R245E mutation alters the heme environment in the distal pocket (Fig. 4*A*). A likely model is that Glu^245^ coordinates the R245E mutant heme iron, as was demonstrated previously in the A264E mutant of *Bacillus megaterium* P450 BM3 (CYP102A1) (21).

Spectral differences are observed between the dithionite-reduced WT and mutant forms of OleT_JE_. For WT OleT_JE_, reduction is associated with a decreased Soret peak intensity and a shift in absorption maximum to 415 nm, with merging of the α and β bands into a broad feature peaking at ∼540 nm (10). Similar spectral shifts occur on the reduction of OleT_JE_ mutants H85Q and F79A (Soret shifts to 416.5 and 418 nm). However, for F79W and F79Y OleT_JE_, the Soret band is red-shifted to ∼422 nm with peak intensity less diminished than in the WT and the H85Q/F79A variants. The red shift suggests that cysteine thiolate protonation occurs in the F79Y/W variants (22). In reduced R245E OleT_JE_, the Soret peak shifts to 417.5 nm, with a Q-band peak at ∼547.5 nm, whereas in reduced R245L the Soret is at 420 nm with α/β bands at 559/536 nm.

Further comparative studies of the heme environments in WT and mutant OleT_JE_ proteins were done using the Fe^II^-CO complexes of these P450s. The WT OleT_JE_ formed predominantly the P450 state with a Soret band at 449 nm and a smaller P420 feature at ∼423 nm. These bands originate from OleT_JE_ Fe^II^-CO complexes in which the proximal ligand is either a cysteine thiolate (P450) or a cysteine thiol (P420) (23, 24). The P450:P420 ratio differs considerably between WT OleT_JE_ and the various mutants. Under the conditions used, the highest P450:P420 ratio occurs for the WT OleT_JE_ with a P450 component of ∼75%. H85Q has a similar proportion of P450 (∼70%) but P450 content decreases in the F79Y mutant (∼45%, similar to the F79A/W mutants), and the R245E mutant forms almost completely the P420 form (∼10% P450) (Fig. 4*B*). These data show that active site mutations influence the OleT_JE_ variant heme environments, including perturbation of the proximal ligand protonation state in the ferrous and/or Fe^II^-CO forms. Although the heme iron of R245E OleT_JE_ is likely coordinated by Glu^245^ carboxylate in the ferric state, CO can clearly displace this distal ligand in the ferrous state of the enzyme. The ferrous R245L/E mutant Soret spectral peaks are similarly positioned at 416.5/417 nm, suggesting displacement of a Glu^245^ distal ligand in the ferrous state for the R245E mutant. The R245L mutant also forms a much higher proportion of the P450 Fe^II^-CO complex at ∼35% (not shown).

##### Interactions of WT and Mutant OleT_JE_ Proteins with Fatty Acid Substrates

To examine the influence of lipid substrates on OleT_JE_ ferric heme iron spin state equilibrium and to determine *K_d_* values for substrate binding, fatty acids of different chain lengths were titrated with WT and mutant OleT_JE_ P450s. For WT OleT_JE_, binding of arachidic acid (C20:0) gives an extensive shift toward HS ferric heme iron, with decreased Soret band intensity at ∼418 nm and increased HS band intensity at 389 nm. Development of HS character is further confirmed by the appearance of a thiolate to HS ferric heme iron charge transfer band at ∼650 nm. For WT OleT_JE_, the amount of HS heme formed decreases as the fatty acid chain length decreases. The *K_d_* values remain low for saturated fatty acids tested in the chain length range from C20 to C14, but affinity decreases considerably for C12 and C10 substrates; *e.g. K_d_* = 1.73 ± 0.08 μm for palmitic acid (C16:0) compared with 47.1 ± 2.1 μm for capric acid (C10:0) (Table 1).

**TABLE 1:** **Binding affinity of saturated fatty acids for WT and mutant forms of OleT_JE_** The dissociation constants (*K_d_* values) for the binding of the saturated fatty acids decanoic acid (capric acid, C10:0), dodecanoic acid (lauric acid, C12:0), tetradecanoic acid (myristic acid, C14:0), hexadecanoic acid (palmitic acid, C16:0), octadecanoic acid (stearic acid, C18:0), and arachidic acid (eicosanoic acid, C20:0) were determined by UV-visible titration with WT OleT_JE_ and with the H85Q, R245E, and F79A/W/Y mutants. NB indicates that no significant binding (indicated by lack of any obvious HS heme iron development) was detected for the indicated mutants. A limit of <2% HS heme iron accumulation is given in these cases. This was also the case for the R245L OleT_JE_ mutant, where no significant optical binding could be detected for any fatty acid tested.

OleT_JE_ variant	Fatty acid *K_d_* (HS)					
	C20:0	C18:0	C16:0	C14:0	C12:0	C10:0
	μ*m* (%)					
WT	4.43 ± 0.19 (95)	4.09 ± 0.16 (70)	1.73 ± 0.08 (25)	1.45 ± 0.05 (45)	11.7 ± 0.43 (40)	47.1 ± 2.1 (35)
H85Q	5.27 ± 0.05 (25)	5.92 ± 0.08 (<10)	5.36 ± 0.18 (<10)	10.5 ± 0.1 (<10)	37.3 ± 2.5 (<10)	NB (<2%)
F79A	3.4 ± 0.3 (<10)	8.7 ± 0.5 (<10)	22.6 ± 1.9 (<10)	39.1 ± 2.5 (<10)	55.0 ± 4.8 (<10)	465 ± 11 (<10)
F79W	4.41 ± 0.27 (20)	4.52 ± 0.20 (15)	12.7 ± 0.7 (<10)	24.1 ± 0.9 (15)	106 ± 9 (<10)	NB (<2%)
F79Y	4.25 ± 0.21 (<10)	8.91 ± 0.33 (<10)	18.2 ± 0.7 (<10)	30.7 ± 1.0 (20)	196 ± 5 (25)	NB (<2%)
R245E	NB (<2%)	NB (<2%)	NB (<2%)	NB (<2%)	NB (<2%)	1260 ± 160 (40)

**FIGURE 5.**
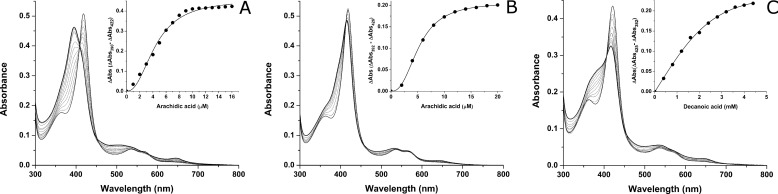
**Fatty acid binding titrations with WT and mutant OleT_JE_ proteins.** *A* shows a UV-visible spectral binding titration for WT OleT_JE_ (5.5 μm) with arachidic acid (C20:0). There is extensive conversion of the LS heme iron (at 418.5 nm) toward HS (at 395 nm). HS heme iron development is also evident from the development of a cysteine thiolate-to-HS ferric heme iron charge transfer species at ∼650 nm. The *inset* shows the fitting of absorbance change *versus* [arachidic acid] using the Hill function, producing an apparent *K_d_* of 4.43 ± 0.19 μm. *B*, shows a titration of the OleT_JE_ H85Q mutant (5.6 μm) with arachidic acid, in this case producing a much smaller spectral conversion toward HS. The *inset* shows the fit of induced absorption change (418.5–415 nm) *versus* [arachidic acid], fitted as above to give a *K_d_* of 5.27 ± 0.05 μm. *C* shows a titration of the OleT_JE_ R245E mutant (4.7 μm) with capric acid (C10:0), with absorption change (∼422.5–384 nm) *versus* [capric acid] data fitted using a hyperbolic function to give a *K_d_* of 1260 ± 160 μm.

Binding of arachidic acid also induces HS heme formation in H85Q and F79A OleT_JE_ mutants but with a much less extensive HS shift than seen in WT OleT_JE_. At apparent saturation with arachidic acid, the Soret band shifts to ∼415 nm for H85Q and to ∼414 nm for F79A OleT_JE_. Arachidic acid spectral binding data for WT and the H85Q OleT_JE_ mutant are compared in (Fig. 5). Despite incomplete HS shifts in these mutants, substrate turnover data confirm that both the H85Q and F79A mutant P450s can oxidize fatty acids in the C10-C20 range (see “Products Formed by Novel OleT_JE_ Mutants” below). Thus, productive substrate binding occurs for these fatty acids in both mutants. These mutations may influence the binding mode of the fatty acid and its carboxylate group, which in turn affects the efficiency with which the substrate can displace the distal water ligand to convert the P450s to a HS state. The reaction of H_2_O_2_ with substrate-bound OleT_JE_ generates reactive heme species (ferryl-oxo and ferryl-hydroxo compounds I and II; Fig. 1) that oxidize the substrate, and the retention of catalytic activity in the H85Q/F79A mutants indicates that fatty acid binding modes remain close to this reactive iron-oxo species such that hydroxylation (at substrate α- and β-carbons) and decarboxylation remain feasible.

It should be noted that for P450 BS_β_ and P450 SP_α_ (where residue 85 is a glutamine), the HS shift upon substrate binding is very small (BS_β_) or not observed at all (SP_α_) (25). The H85Q OleT_JE_ mutant thus mimics the spectral properties of these other bacterial peroxygenases on binding fatty acid substrates. For both the H85Q and F79A OleT_JE_ mutants, there is a pattern of increasing substrate *K_d_* values as the fatty acid chain length decreases, consistent with the properties of WT OleT_JE_ and likely reflecting the fewer hydrophobic interactions made by shorter chain substrates in the active site channel (Table 1).

The F79W and F79Y OleT_JE_ proteins show modest spin state shifts with most fatty acids tested, suggesting again that the binding modes of these substrates are perturbed. However, in the F79Y OleT_JE_, there is a notable increase in HS content on saturation with lauric acid (C12:0, ∼25% high spin) and myristic acid (C14:0, ∼20% high spin) compared with longer chain fatty acids (<10% for arachidic acid, stearic acid (C18:0) and palmitic acid). This suggests that these shorter chain fatty acids can adopt binding modes in F79Y OleT_JE_ that are compatible with more efficient displacement of the distal water molecule than can occur with longer chain substrates, probably in part because of their greater solubility. With the exception of the R245E OleT_JE_ variant, the *K_d_* values for the longest substrates (C20:0 and C18:0) are similar for the WT and mutant OleT_JE_ enzymes (Table 1). As fatty acid chain length decreases from C16:0, there are wider variations in the substrate *K_d_* values, with all mutants apart from R245E (see below) showing weaker binding of fatty acids from C16:0 to C10:0 by comparison with the WT OleT_JE_.

In the case of the R245L mutant, addition of the solvents ethanol, methanol, and DMSO resulted in the development of up to ∼10% HS heme iron. This phenomenon was not observed with the R245E mutant, likely because of the Glu^245^ carboxylate occupying the distal ligand position on the heme iron. This issue prevented any accurate assessment of the role of fatty acids in perturbing the R245L heme iron spin state equilibrium, because spectral changes induced by fatty acids dissolved in these solvents were of similar magnitude to those induced using solvents alone. However, addition of 1 mm lauric acid prepared in aqueous buffer A produced no significant change in the R245L UV-visible spectrum. Similarly, addition of a saturating amount of arachidic acid (in ethanol) did not induce any further R245L HS heme iron development over that caused by solvent alone. Thus, fatty acid substrate binding modes in this mutant may be particularly inefficient in displacement of the axial water ligand in ferric R245L OleT_JE_.

The R245E OleT_JE_ mutant exhibits unusual behavior in that there is no apparent shift in Soret maximum from 423 nm or development of HS heme iron observed in optical titrations with any of the fatty acids from C20:0 to C12:0. However, titration with capric acid (C10:0), resulted in ∼40% HS heme iron accumulation at apparent saturation (Fig. 5*C*). These observations suggest that the longer chain fatty acids do not bind R245E OleT_JE_ in a mode that enables HS development/displacement of a Glu^245^ axial ligand. However, the substantial HS heme accumulation in R245E OleT_JE_ on binding capric acid suggests that this short chain substrate can displace the axial ligand, albeit with a very high *K_d_* (1260 ± 160 μm). Coordination of heme iron by glutamate oxygen was also observed in the P450 BM3 A264E mutant, with arachidonic acid substrate binding causing nearly complete distal coordination by the Glu^264^ carboxylate (21, 26). However, the situation may be reversed for the OleT_JE_ R245E mutant, with capric acid partially displacing a glutamate ligand at high concentrations.

##### Stopped Flow Kinetic Analysis of Fatty Acid Oxidation by WT and Mutant OleT_JE_ P450s

Stopped flow absorption spectroscopy was used to probe the effects of mutations to His^85^ (H85Q) and Phe^79^ (F79A/W/Y) on the kinetics of OleT_JE_ single turnover substrate oxidation reactions. WT, H85Q, and F79A/W variants were incubated with arachidic acid, whereas F79Y OleT_JE_ was incubated with nearly saturating lauric acid to achieve the maximal heme iron spin state conversion for this mutant. The H_2_O_2_-dependent oxidation of substrates was observed by the rapid HS to LS conversion of the OleT_JE_ heme iron in each case. Reaction progress was monitored at wavelengths close to the ferric, LS Soret maximum for WT OleT_JE_ and the four mutants. Rate constants for the single turnover reactions (*k*_obs_) were determined using H_2_O_2_ concentrations up to 200 μm. Under the conditions used, plots of *k*_obs_ *versus* [H_2_O_2_] were hyperbolic for WT OleT_JE_ and for the H85Q and F79Y variants. The data were fitted using a hyperbolic function to derive the limiting rate constant (*k*_lim_) and the apparent *K_d_* for H_2_O_2_. The WT OleT_JE_ had values of *k*_lim_ = 115 ± 8 s^−1^, *K_d_* = 107 ± 16 μm, compared with 101 ± 16 s^−1^, 749 ± 141 μm for F79Y, and 286 ± 18 s^−1^, 327 ± 29 μm for H85Q. The dependence of *k*_obs_ *versus* [H_2_O_2_] was linear for the F79A and F79W mutants, with second order rate constants of 0.188 ± 0.004 μm^−1^ s^−1^ and 0.107 ± 0.005 μm^−1^ s^−1^, respectively. The comparable second order rate constants (*k*_lim_/*K_d_*) for the other proteins are 1.075 ± 0.236 μm^−1^ s^−1^ (WT), 0.875 ± 0.133 μm^−1^ s^−1^ (H85Q), and 0.838 ± 0.507 μm^−1^ s^−1^ (F79Y).

Stopped flow kinetic data for the reactions of WT and mutant forms of fatty acid-bound OleT_JE_ with H_2_O_2_ indicate that the WT, H85Q, and F79Y enzyme single turnovers are more efficient than are those of the other two Phe^79^ mutants. Although this is consistent with the superior catalytic properties of the WT and H85Q mutants in product formation (Table 3), the F79A OleT_JE_ is a more productive enzyme for C10–C16 substrates than are F79Y/W, with more similar levels of product formation for the F79A/Y/W mutants with C18 and C20 substrates. However, in view of only minor development of HS heme iron in arachidic acid-bound F79Y OleT_JE_, lauric acid was used instead with the F79Y variant to enhance HS formation and to improve experimental data quality. In this case it appears that single turnover efficiency (*i.e.* the apparent second order rate constant for F79Y OleT_JE_-dependent substrate oxidation by H_2_O_2_) is at a level similar to that of the WT and H85Q OleT_JE_ enzymes with arachidic acid. Despite this finding, percentage product formation for lauric acid with the F79Y mutant is much lower than observed for the WT/H85Q/F79A OleT_JE_ enzymes with the same substrate and similar to that for the F79A OleT_JE_ with arachidic acid. These data suggest that other factors (*e.g.* the rate constants for substrate association/product dissociation and/or efficiency of substrate oxidation with different chain substrates) have a strong influence on overall product formation.

##### Characterization of OleT_JE_ WT and Mutant P450 Heme Coordination by EPR

**FIGURE 6.**
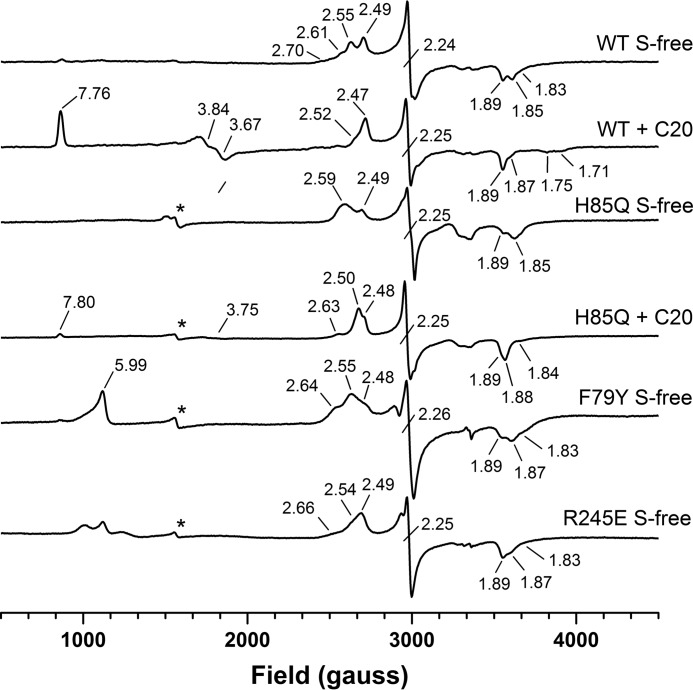
**EPR spectroscopic properties of WT and mutant forms of OleT_JE_.** X-band EPR spectra are shown for WT and H85Q OleT_JE_ mutants in both ligand-free and arachidic acid substrate-bound (C20:0) forms and for the F79Y and R245E OleT_JE_ mutants in their ligand-free forms. The WT and mutant forms of OleT_JE_ have complex LS EPR spectra with several distinct species (see Table 2 for sets of *g* values). HS heme iron accumulation is seen following addition of arachidic acid to WT OleT_JE_ (*g* = 7.76/3.84/1.71 and 7.76/3.67/1.75), but only a very small HS signal is seen for the arachidic acid-bound H85Q mutant (*g* = 7.80/3.75). The *asterisks* show signals for a proportion of a HS penta-coordinated, thiol-ligated, ferric heme species in the H85Q, F79Y, and R245E mutants.

Continuous wave X-band EPR spectra were recorded for WT and all mutant forms of OleT_JE_ in their substrate-free and arachidic acid-bound states to characterize the heme coordination state and the influence of mutations to the key residues in the heme distal pocket. As reported previously, the substrate-free form of WT OleT_JE_ has a complex EPR spectrum with at least four LS (*S* = 1/2) ferric species with rhombic anisotropy; the most prominent of which have *g* values of *g*_z_ = 2.49, *g*_y_ = 2.24, and *g*_x_ = 1.89 (2.49/2.24/1/89) and 2.55/2.24/1.85 (Fig. 6) (10). The *g* values for these species and those for all other mutants and their substrate-bound forms are presented in Table 2. These LS species are consistent with ferric heme iron coordinated by cysteine thiolate and a distal water ligand in a large water-filled active site, and with there being a variety of coordination geometries and hydrogen bonding interactions/networks with the distal water ligand. There is no major contribution from a HS (S = 5/2) species in the WT substrate-free form, but a substantial HS component appears on the addition of arachidic acid to WT OleT_JE_. This is split into two major species with *g* values of 7.76/3.84/1.71 and 7.76/3.67/1.75. As discussed previously, this heterogeneity likely results from, for example, changes in conformation of the heme and thiolate ligand geometry, rather than from any axial ligand switch in the HS, pentacoordinate state (10). The binding of arachidic acid also results in a dominant LS form with *g* values of 2.47/2.25/1.89, suggesting a much more ordered active site organization in the substrate-bound state.

**TABLE 2:** **EPR *g* values for substrate-free and fatty acid-bound forms of WT and mutant OleT_JE_ proteins** X-band EPR data were collected at 10 K for the substrate-free and arachidic acid-bound forms of OleT_JE_. The *g* values for the LS species and for HS species (where evident) are presented. In the case of fatty acid-bound forms giving weak HS signals, only those component *g* values that can be accurately identified are shown. ND indicates not determined in those cases where no significant HS ferric heme iron signal could be identified or where signals were of insufficient intensity to be accurately assigned. For each of the OleT_JE_ LS forms, two to four different rhombic species are identified in each case. The most prominent of these species is iidentified as the “Major species” in each case.

OleT_JE_ variant	Rhombic HS signals	Rhombic LS signals (*g*_z_/*g*_y_/*g*_x_)	
		Major species	Minor species
WT	ND	2.49/2.24/1.89	2.70/2.24/1.83; 2.61/2.24/1.85; 2.55/2.24/1.85
H85Q	ND	2.59/2.25/1.85	2.49/2.25/1.89
F79A	ND	2.48/2.25/1.89	2.65/2.25/1.84; 2.58/2.25/1.86
F79W	ND	2.54/2.25/1.86	2.65/2.25/1.84; 2.47/2.25/1.89
F79Y	ND	2.55/2.26/1.87	2.64/2.26/1.83; 2.48/2.26/1.89
R245L	ND	2.54/2.25/1.87	2.65/2.25/1.85; 2.46/2.25/1.90
R245E	ND	2.49/2.25/1.89	2.66/2.25/1.83; 2.54/2.25/1.87
WT+C20:0	7.76/3.84/1.71; 7.76/3.67/1.75	2.47/2.25/1.89	2.52/2.25/1.87
H85Q+C20:0	7.80/3.75	2.50/2.25/1.88	2.63/2.25/1.84; 2.48/2.25/1.89
F79A+C20:0	ND	2.49/2.26/1.89	2.60/2.26/1.84; 2.54/2.26/1.86
F79W+C20:0	ND	2.54/2.25/1.86	2.67/2.25/1.83; 2.46/2.25/1.89
F79Y+C20:0	7.79	2.56/2.26/1.86	2.65/2.26/1.83; 2.49/2.26/1.89
R245L+C20:0	7.70, 3.74	2.53/2.25/1.87	2.65/2.25/1.85; 2.46/2.25/1.90
R245E+C20:0	ND	2.53/2.25/1.87	2.67/2.25/1.85; 2.47/2.25/1.90

For the H85Q OleT_JE_ mutant, the substrate-free LS spectrum is less complex than that for WT OleT_JE_. There are two major LS species at 2.59/2.25/1.85 and 2.49/2/25/1.89. The signal for the former is broad and may encompass two LS species. The addition of arachidic acid produces a much smaller HS signal than in WT OleT_JE_ (with features at *g*_z_ = 7.8 and *g*_y_ = 3.75) and alters the LS spectrum, with two major species at 2.50/2.25/1.88 and 2.48/2.25/1.89. The LS heme iron signals for the H85Q mutant are quite distinct from those for WT OleT_JE_ in both the substrate-free and arachidic acid-bound forms, indicating that the H85Q mutation alters the environment of the distal pocket around the heme iron, *e.g.* through altering the hydrogen bonding network to the distal water ligand in the LS substrate-free form (Fig. 6).

In F79A OleT_JE_ the major LS species is at 2.48/2.25/1.89, similar to that of the main LS species in the WT enzyme. Minor species are at 2.65/2.25/1.84 and 2.58/2.25/1.86. The F79Y and F79W mutants have similar spectra to F79A OleT_JE_, although the central set of *g* values is predominant in these mutants (2.55/2.26/1.87 and 2.54/2.25/1.86, respectively). Binding of arachidic acid does not produce any significant HS signal in the F79A/W mutants, although a small HS signal is observed for substrate-bound F79Y OleT_JE_ (*g*_z_ = 7.79). However, substrate does cause some perturbations to the Phe^79^ mutants LS *g* values, particularly in the case of the F79A variant (Table 2). Notably, there is a large axial HS signal at *g* = 5.99 in both the F79W and F79Y OleT_JE_ mutants that likely relates to HS penta-coordinated, thiol-ligated, ferric heme species in these mutants. This observation is consistent with some loss of heme in these variants, and UV-visible studies of these mutants also revealed a propensity for cysteine thiolate protonation of their heme iron to form the P420 state.

The R245E/L mutants result in less complex substrate-free EPR spectra than in WT OleT_JE_, with two major, similar sets of *g* values for both R245E (2.54/2.25/1.87 and 2.49/2.25/1.89) and R245L (2.54/2.25/1.87 and 2.46/2.25/1.90). The addition of arachidic acid has little effect on these LS *g* values but does produce a small rhombic HS signal (*g*_z_ = 7.70, *g*_y_ = 3.74) in the R245L mutant that mirrors the data from UV-visible titrations and results from effects of the solvent (Fig. 6 and Table 2). The R245E-capric acid complex proved unstable at the high protein concentration required for EPR analysis, aggregating and precipitating at ∼50 μm.

##### Products Formed by Novel OleT_JE_ Mutants

Fatty acid substrate conversion reactions were set up using 1 μm WT, H85Q, F79A/W/Y, and R245L/E OleT_JE_ enzymes and using 200 μm fatty acids (capric acid, lauric acid, myristic acid, palmitic acid, stearic acid, or arachidic acid), as described under “Experimental Procedures.”

**FIGURE 7.**
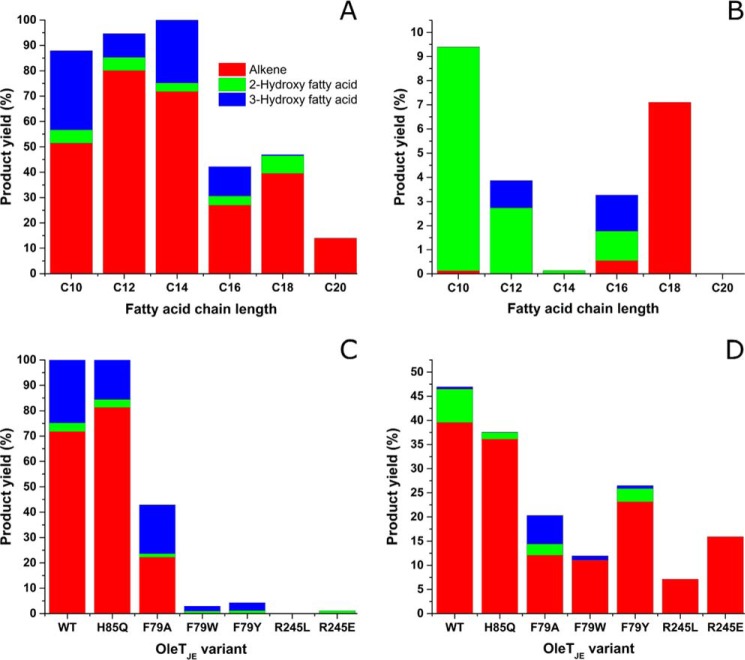
**Products of fatty acid turnover from WT and mutant OleT_JE_ enzymes.** The bar charts show yields of different alkenes (*red*), 2-hydroxy fatty acids (*green*), and 3-hydroxy fatty acids (*blue*) resulting from catalytic turnover by WT and mutant forms of OleT_JE_, as described under “Experimental Procedures.” The data shown are averages from two sets of experiments, with values varying by <10% in all cases. *A* shows proportions of the different products formed in turnover of C10:0, C12:0, C14:0, C16:0, C18:0, and C20:0 substrates by WT OleT_JE_. *B* shows the products formed by the R245L OleT_JE_ mutant with the same substrates, illustrating a switch from fatty acid hydroxylation toward fatty acid decarboxylation (terminal alkene formation) at longer substrate chain lengths. *C* shows products formed from the C14:0 substrate (myristic acid) by WT and all OleT_JE_ mutants. *D* shows products formed from the C18:0 substrate (stearic acid) by WT and all OleT_JE_ mutants.

For WT OleT_JE_, activity was highest for shorter chain length substrates (C10:0 to C14:0), with essentially complete oxidation of myristic acid observed. These data are consistent with studies by Liu *et al* (12). Activity decreased with longer chain lengths, possibly because of the lower solubility of the substrates and/or the higher affinity of the terminal alkenes/hydroxylated products formed for the OleT_JE_ active site (15). For all the fatty acids tested with WT OleT_JE_ (C10:0 to C20:0), the major reaction was decarboxylation of the fatty acids to form terminal alkenes. For most substrates, 3-hydroxy fatty acid products were more prevalent than 2-hydroxy fatty acids. However, the reverse was the case for stearic acid (C18:0). When arachidic acid was used as a substrate, only 1-nonadecene was detected, with negligible amounts of 2-/3-hydroxy arachidic acid produced (Fig. 7*A* and Table 3).

**TABLE 3:** **Conversion of fatty acid substrates into alkene and hydroxylated fatty acid products by WT and mutant OleT_JE_ enzymes** The WT, H85Q, R245E/L, and F79A/W/Y OleT_JE_ enzymes were used to catalyze H_2_O_2_-dependent decarboxylation/hydroxylation of saturated fatty acid substrates (C10:0-C20:0) as described under “Experimental Procedures.” The percentage conversions of each fatty acid substrate into their respective (i) *n*-1 terminal alkene, (ii) 2-OH fatty acid, and (iii) 3-OH fatty acid products are shown for each OleT_JE_ variant. The percentage of the various products formed in each reaction is given to the nearest 1% where these products form 10% or more of the total and to the nearest 0.1% where products form less than 10% of the overall yield. The total product formation for each OleT_JE_ variant/substrate combination is presented in the same way. The data presented are average values from two sets of experiments differing by less than 10% in all cases.

Substrate	Products	OleT_JE_ variant and % products formed						
		WT	H85Q	F79A	F79W	F79Y	R254L	R245E
C10:0	1-Nonene	51	65	37	1.5	0.3	0.1	0.2
	2-OH capric acid	5.2	8	1.4	2.1	0	9.3	0
	3-OH capric acid	31	25	15	2.1	0	0	0
	Total conversion	87	98	53	5.7	0.3	9.4	0.2
C12:0	1-Undecene	80	79	64	1.3	1.3	0	0
	2-OH lauric acid	5.2	7.8	7.4	3.6	3.4	2.7	2.6
	3-OH lauric acid	9.0	9.2	8.5	1.6	1.4	1.1	1.1
	Total conversion	94	96	80	6.5	6.1	3.8	3.7
C14:0	1-Tridecene	72	81	22	0	0	0	0
	2-OH myristic acid	3.3	3.1	1.4	1.0	1.2	0.1	1.1
	3-OH myristic acid	25	16	19	2.0	3.1	0	0
	Total conversion	100	100	42	3.0	4.3	0.1	1.1
C16:0	1-Pentadecene	27	22	9.3	1.2	0.8	0.5	0.6
	2-OH palmitic acid	3.6	3.2	2	2	1.6	1.2	1.2
	3-OH palmitic acid	12	13	5.1	2.3	1.7	1.5	1.5
	Total conversion	43	38	16	5.5	4.1	3.2	3.3
C18:0	1-Heptadecene	40	36	12	11	23	7.1	16
	2-OH stearic acid	7	1.4	2.3	0	2.7	0	0
	3-OH stearic acid	0.4	0.1	5.9	0.9	0.7	0	0
	Total conversion	47	38	20	12	27	7.1	16
C20:0	1-Nonadecene	14	11	5	3.4	2.5	0	0.1
	2-OH arachidic acid	0	0	0	0	0	0	0
	3-OH arachidic acid	0	0	0	0	0	0	0
	Total conversion	14	11	5	3.4	2.5	0	0.1

OleT_JE_ His^85^ was postulated as a proton donor to iron-oxo species in the WT OleT_JE_ reaction (10). The P450 BS_β_ and SP_α_ enzymes have a glutamine at this position, and catalyze predominantly (BS_β_) or exclusively (SP_α_) fatty acid hydroxylation. The H85Q mutant was found to generate a product profile very similar to that for the wild type OleT_JE_ for all substrates tested (Fig. 7*B* and Table 3). The *n* − 1 terminal alkenes are again the dominant products in all cases, with 3-hydroxy fatty acids formed to greater extents than 2-hydroxy fatty acids for substrates from C10:0-C16:0. As with WT OleT_JE_, the 2-hydroxy stearic acid product is formed to higher levels than 3-hydroxy stearic acid, and 1-nonadecene is the only significant product from arachidic acid with H85Q OleT_JE_.

The F79A OleT_JE_ mutant exhibited decreased activity compared with the WT and H85Q enzymes for all fatty acids tested. F79A OleT_JE_ mutant activity was highest with lauric acid, with 80% product conversion (compared with 94%/96% for WT/H85Q OleT_JE_), but overall product formation was much lower with myristic acid (42% for F79A compared with 100% with WT/H85Q). The F79A OleT_JE_ mutant produced 3-hydroxy fatty acids in excess over 2-hydroxy fatty acids for all substrate chain lengths from C10-C18. For arachidic acid, the only product formed was 1-nonadecene, albeit with only 5% conversion (compared with 14%/11% for WT/H85Q OleT_JE_) (Table 3).

The F79W/Y mutants were generated to analyze effects of potentially more conservative mutations to Phe^79^. However it was found that, with one exception, both the F79W/Y OleT_JE_ mutants had much lower activity than F79A OleT_JE_. The only outlier was the F79Y mutant with stearic acid substrate, where overall product formation was 27% compared with 20% for F79A OleT_JE_, because of a higher amount of 1-heptadecene produced by the F79Y mutant. Although amounts of alkene products from the F79A/W/Y mutants are broadly comparable for the C18:0 and C20:0 substrates, alkenes are formed in much lower amounts in the F79W/Y mutants compared with the F79A OleT_JE_ for the C10:0-C16:0 substrates (Table 3).

Crystallographic data indicated that OleT_JE_ Arg^245^ plays a key role in catalysis by coordinating the carboxylate group of the fatty acid substrate (10). The R245L and R245E OleT_JE_ mutants were generated and purified to investigate effects of side chain charge removal and reversal. The R245L/E OleT_JE_ mutants generally have much lower levels of activity than WT OleT_JE_ and the two most productive mutants (H85Q and F79A). However, the R245L/E mutants convert 7.1%/16% stearic acid to 1-heptadecene, with no detectable hydroxylated products. Almost no decarboxylated product (1-nonadecene) was observed for these mutants with arachidic acid, and alkene production was in the range from 0 to 0.6% for the R245L/E mutants and C10:0-C16:0 substrates (Table 3). The unusual “switch” from mainly substrate hydroxylase activity at shorter chain lengths toward decarboxylase activity for stearic acid is shown in Fig. 7*C*, and Fig. 7*D* shows the product profiles from WT and all OleT_JE_ mutant enzymes with stearic acid (C18:0) substrate. Alkenes are the major products formed by WT and all OleT_JE_ mutants with this substrate.

##### Structural Properties of OleT_JE_ Mutant Enzymes

Crystallization trials were undertaken for all OleT_JE_ mutant enzymes. Crystals were obtained for both the H85Q and F79A mutant P450s in complex with arachidic acid substrate, as well as for the WT OleT_JE_ in conditions containing formate. In the latter case, this produced a complex in which formate coordinates the WT OleT_JE_ heme iron in the distal position. Structures for these mutant enzymes were determined to 1.44 Å (WT/formate), 1.80 Å (H85Q/arachidic acid) and 1.95 Å (F79A/arachidic acid) (Table 4). Attempts to crystallize other mutants were not successful, largely because of the propensity of other OleT_JE_ mutants to aggregate at high concentrations.

**TABLE 4:** **Data reduction and final structural refinement statistics for OleT_JE_ WT and mutant crystal structures** The data are presented for WT OleT_JE_ bound to formate and for the arachidic acid-bound forms of the H85Q and F79A OleT_JE_ mutants.

	OleT_JE_ WT (formic acid)	OleT_JE_ H85Q (C20:0)	OleT_JE_ F79A (C20:0)
**Data collection**			
Space group	P4_3_2_1_2	P2_1_2_1_2_1_	P2_1_2_1_2_1_
Cell dimensions			
*a*, *b*, and *c* (Å)	60.170, 60.170, 241.70	48.928, 115.494, 163.247	51.88, 110.120, 164.850
α, β, γ (°)	90, 90, 90	90, 90, 90	90, 90, 90
*R*_merge_ (%)	0.035 (0.625)	0.051 (0.681)	0.057 (0.842)
*I*/σ*I*	25.8 (2.9)	11.4 (2.6)	8.6 (1.8)
Completeness (%)	99.9 (100.0)	99.7 (99.6)	99.6 (99.8)
Redundancy	12.8 (6.5)	6.1 (5.2)	6.5 (6.6)

**Refinement**			
Resolution (Å)	48.21–1.44 (1.47–1.44)	25.50–1.80 (1.83–1.80)	55.06–1.95 (2.00–1.95)
No. reflections	77,736 (5628)	81,942 (5951)	66,360 (4861)
*R*_work_/*R*_free_	13.46/17.27 (21.4/28.0)	18.27/21.99 (25.7/29.3)	15.72/20.12 (27.1/33.1)
No. non-hydrogen atoms	3798	7482	7704
Mean B factor (A^2^)	19.79	27.35	29.706
R.m.s. deviations			
Bond lengths (Å)	0.020	0.019	0.013
Bond angles (°)	2.020	1.875	1.270

**PDB numbers**	**5M0N**	**5M0O**	**5M0P**

**FIGURE 8.**
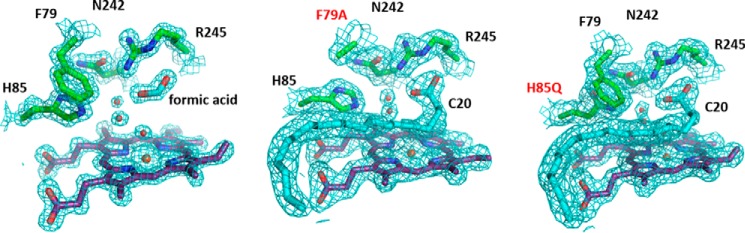
**Active site structures for WT and mutant OleT_JE_ proteins.** The three panels show active site details from the crystal structures of WT OleT_JE_ in complex with formic acid (*left panel*) and for the F79A (*middle panel*) and H85Q (*right panel*) OleT_JE_ mutants bound to arachidic acid. Selected residues in the active site are shown as atom colored sticks (*green carbons*), whereas the heme group and associated ligands are shown with *purple* and *cyan* carbons, respectively. Positions of mutations are labeled in *red*. Electron density is shown as a *blue mesh* contoured at 2 σ for the WT OleT_JE_-formic acid complex, and at 1.5 σ for the two mutant structures. Three water molecules are clearly defined in the active site of these three structures (oxygens shown as *red spheres*), one of which acts as the sixth ligand to the heme iron.

Few structural changes occur in the OleT_JE_ structure because of the mutations. Arachidic acid substrate is bound similarly in both the H85Q and F79A mutants as was previously reported for the WT OleT_JE_ (10). However, in the WT arachidic acid-bound structure, a sixth ligand water molecule could not be distinguished, likely because of the HS state of the WT OleT_JE_ substrate-ligand complex. In contrast, for both OleT_JE_ H85Q/F79A mutant structures, a sixth ligand water molecule is clearly visible. A similar situation is observed for the higher resolution structure of the WT OleT_JE_-formic acid complex, which remains LS in solution (Fig. 8). UV-visible substrate binding data for the H85Q/F79A mutants indicate much smaller HS shifts than observed for WT OleT_JE_, consistent with retention of the aqua-ligated LS state in the major fraction of the mutant P450s in the crystal structures.

The lack of any large scale structural changes in the H85Q/F79A OleT_JE_ mutants suggests that that differences in product profiles for the WT and various OleT_JE_ mutants are linked to subtle changes in the active site, possibly related to mobility of active site residues and/or substrates. Minor changes in the positioning and dynamic behavior of the α and β substrate hydrogens might have substantial effects on the product profile but are unlikely to be distinguished using medium resolution crystallography.

## Discussion

The peroxygenase enzyme P450 BS_β_ (CYP152A1) was discovered in the late 1990s by Matsunaga *et al.* (27) and shown to catalyze the H_2_O_2_-dependent 1-(α) and 2-(β) hydroxylation of myristic acid, with 2-hydroxy myristic acid being the main product. Its counterpart P450 SP_α_ (CYP152B1) was also identified by Matsunaga *et al.* and shown to catalyze the α-hydroxylation of lipids, producing 2-hydroxy myristic acid from the C14:0 substrate, as well as catalyzing 2-hydroxylation of longer chain saturated fatty acids and mono-unsaturated fatty acids (28, 29). However, only after studies by Rude *et al.* (9) was the capacity revealed of the related OleT_JE_ enzyme (CYP152L1) to oxidatively decarboxylate fatty acids to form terminal alkenes. They also demonstrated that 1-pentadecene was formed not only by H_2_O_2_-dependent oxidative decarboxylation of palmitic acid in OleT_JE_ but also by P450 BS_β_ and related P450s from *Corynebacterium efficiens*, *Kocuria rhizophila*, and *Methylobacterium populi* (9, 30). OleT_JE_ is the most productive of the fatty acid peroxygenase P450s characterized to date with respect to its generation of alkene products over hydroxylated fatty acids. OleT_JE_ was also shown to be functional in alkene production when driven by a redox partner system or using a photocatalytic system to generate H_2_O_2_ (11, 14). OleT_JE_ is currently the most studied of the peroxygenase P450s, and the one with greatest potential for biotechnological applications in production of alkenes for use in biofuel production, for example (31).

The determination of the crystal structure of OleT_JE_ revealed a high level of similarity with P450 BS_β_ in overall fold and active site structure (10). The OleT_JE_ binding pocket is more extended than that of P450 BS_β_, explaining its ability to accommodate fatty acids as long as arachidic acid (C20:0). The OleT_JE_ structure highlights structural features common to other P450s (including the heme binding motif surrounding the conserved cysteine thiolate), as well as key residues involved in interacting with the substrate carboxylate. Amino acid alignment of OleT_JE_ with other characterized P450 peroxygenases reveals that only 48 amino acids are in conserved positions in each of the seven P450 enzymes aligned, which vary in length from 415 to 428 amino acids (Fig. 2). The substrate carboxylate coordinating OleT_JE_ Arg^245^ and its adjacent Pro^246^ are conserved in all these peroxygenases, replacing the acid-alcohol pair (*e.g.* Asp^251^/Thr^252^ in P450cam) found in most mono-oxygenase P450s and involved in protonation of ferric-peroxo and ferric-hydroperoxo catalytic cycle intermediates (3). These key mutations illustrate how these peroxygenases have diverged from mono-oxygenase P450s, exchanging a dipeptide involved in iron-oxo species protonation for one selected for binding the substrate carboxylate. The R245E/L mutants are severely catalytically compromised, consistent with a crucial role of Arg^245^ in orientating fatty acid carboxylate into a position compatible with efficient decarboxylation/hydroxylation of the substrate. However, these mutants do form small amounts of products (Table 3). The R245L/E mutants produced most alkene from the C18:0 substrate stearic acid but negligible amounts from the C20:0 substrate arachidic acid, potentially because of tighter product binding for the longer chain substrate. With one exception, the R245L/E mutants do not exhibit any significant substrate-induced HS ferric heme iron development. Only in the case of R245E with the C10:0 substrate capric acid is there any substantial HS accumulation (∼40%), albeit achieved at high substrate concentration (*K_d_* = 1260 ± 160 μm) (Table 1). The HS conversion suggests that capric acid does displace Glu^245^ from a distal position on the heme iron, although only a small proportion of the substrate is converted to 1-nonene (0.2%), and no significant formation of hydroxylated forms of capric acid was observed (Fig. 5*C* and Table 3).

The H85Q mutant was generated to explore the role of the OleT_JE_ His^85^ residue, which is replaced by a glutamine in the well characterized P450 BS_β_, P450 SP_α_ and *Clostridium acetobutylicum* P450 CLA (CYP152A2) enzymes, all of which catalyze predominantly or exclusively fatty acid hydroxylation (33) (Fig. 2). The OleT_JE_ H85Q mutant gives a very similar product profile to WT OleT_JE_ with each substrate tested. However, UV-visible binding studies demonstrate that substrate-induced H85Q HS heme accumulation is much lower than in the WT OleT_JE_ in all cases (Tables 1 and 3). EPR studies confirms much lower accumulation of HS heme iron in arachidic acid-bound H85Q OleT_JE_ compared with the WT enzyme (Fig. 6). However, heme spectral shifts are sufficient to determine *K_d_* values for all substrates with H85Q OleT_JE_, showing that affinity is similar to WT OleT_JE_ for longer chain substrates but significantly weaker for myristic acid and lauric acid substrates, with negligible heme spin state change observed on titration of H85Q OleT_JE_ with capric acid (Table 1). The substantially diminished (or completely absent) extents of substrate-dependent HS shift in the H85Q mutant are consistent with the properties of the SP_α_, BS_β_, and CLA peroxygenases (18–20, 33). The similarity in product profiles between WT and H85Q OleT_JE_ suggests that His^85^ is not crucial for proton transfer to transient heme iron-oxo species to facilitate C–C bond scission and alkene production or that alternative pathway(s) exist in the H85Q mutant. A similar conclusion regarding the non-essentiality of a histidine in this position was also reached by Amaya *et al.* (30) in their studies of the *M. populi* OleT_JE_ ortholog (CYP-MP), in which a methionine is located in the relevant position, although the CYP-MP enzyme is a less efficient decarboxylase than OleT_JE_.

OleT_JE_ structural data showed that the His^85^ imidazole moiety is “sandwiched” between Phe^79^ and the heme edge (10). The F79A/Y/W mutants were produced and characterized to explore the effects of both removing the aromatic side chain and extending its size and chemical character. All these mutants showed reduced total activity compared with WT and H85Q OleT_JE_, with F79A OleT_JE_ being the most active, forming between 36 and 43% of the product amount from WT OleT_JE_ in the same unit time for the C14:0-C20:0 substrates but increasing to 61 and 85% of the amount for the shorter chain capric acid and lauric acid substrates. The alkenes are the major products with F79A OleT_JE_ and all substrates, with the 3-hydroxylated fatty acid formed in excess over 2-hydroxyated fatty acids for the C10-C18 substrates. For the C20:0 arachidic acid, only 1-nonadecene was formed to a detectable level at 5% of the starting material (Table 3).

Despite the more “conservative” nature of the F79W and (particularly) the F79Y mutations, the activities of these variants were much lower than observed for F79A OleT_JE_ with C10:0-C16:0 substrates. Within this group of substrates, hydroxylated fatty acids are formed in excess over alkenes in all cases except for the F79Y mutant with capric acid (where 1-nonene is the only product observed at 0.3% of the starting material), and total product formation does not exceed 6.5% for the F79Y/W mutants. However, with the C18:0 substrate stearic acid, the major product is 1-heptadecene (23%/11% products, respectively), and 1-nonadecene is the only product from arachidic acid (2.5%/3.4% products, respectively).

As is also the case for the H85Q mutant, each of the F79A/Y/W mutants give less substantial HS heme conversions than seen in WT OleT_JE_ (Table 1). All Phe^79^ mutants also show a similar pattern of decreasing affinity as fatty acid chain length decreases, with capric acid not inducing any significant heme spin state change in the F79Y/W OleT_JE_ mutants.

Many bacterial P450s exploit a substrate binding-dependent LS to HS heme shift to regulate catalytic activity through development of a more positive potential in the substrate-bound form of the heme iron. For example, arachidonic acid binding to P450 BM3 causes an ∼140-mV increase in ferric heme iron reduction potential, facilitating electron transfer from the linked cytochrome P450 reductase domain to drive catalysis (34). However, OleT_JE_ shows negligible change in the redox potential of the heme Fe^III^/Fe^II^ couple on arachidic acid binding (−105 mV and −103 mV *versus* normal hydrogen electrode, respectively, for substrate-free and substrate-bound forms), consistent with its dependence on H_2_O_2_ for generating reactive iron-oxo species (10). The relatively positive potential in OleT_JE_ is likely related in part to the structural arrangement around the proximal cysteinate ligand. Here, it is notable that a phenylalanine residue (typically seven amino acids before the cysteinate) heavily conserved in mono-oxygenase P450s is absent in all of the peroxygenases aligned in Fig. 2. Mutations to the relevant Phe^393^ residue in P450 BM3 cause substantial changes in heme iron potential (35, 36). Recent studies showed that OleT_JE_ turnover can be driven by heterologous redox partner systems, and this is likely facilitated by the large driving force (>210 mV) for electron transfer from NAD(P)H through redox partner proteins and onto the substrate-bound OleT_JE_ heme iron (11, 12).

In conclusion, we provide a detailed study of the properties of WT OleT_JE_ and of important active site mutants of this alkene producing P450. We show that glutamine substitutes effectively for histidine in the OleT_JE_ H85Q mutant, which has very similar product profiles to WT OleT_JE_. The major difference lies in the much weaker ability of fatty acid substrates to induce HS heme iron formation in the H85Q mutant (Fig. 5*B*), a property consistent with those of the P450 SP_α_ and P450 BS_β_ peroxygenases, both of which have a glutamine in the same position (20, 38). The data appear to rule out a key role for His^85^ in proton donation to reactive iron-oxo species in OleT_JE_, and suggest that the system is robust and that alternative protonation pathway(s) may exist. Mutations to OleT_JE_ Phe^79^ also diminish substrate-dependent heme HS conversion and weaken affinity for shorter chain (C10:0-C16:0) fatty acid substrates. This translates into much lower product formation for the F79Y/W mutants from these substrates, although F79A OleT_JE_ is less severely affected, with between 36 and 85% overall product formation compared with that of WT OleT_JE_ for C10:0-C20:0 substrates (Table 3). These data indicate that the more “conservative” F79Y/W mutations are actually more disruptive to active site structure and catalytic efficiency than is the F79A mutation, a conclusion consistent with the greater propensity of the F79Y/W mutants to aggregate/precipitate and their failure to crystallize. These properties are shared with the R245L/E variants, in which the substrate carboxylate tethering Arg^245^ is mutated. Each of the F79Y/W and R245E/L mutations show some heme depletion, with R245E OleT_JE_ being the worst affected (Fig. 4*B*). The red-shifted (423 nm) Soret band of the oxidized R245E OleT_JE_ protein suggests that the glutamate may coordinate the heme iron in the ferric state. The R245L/E mutants retain low activity, with stearic acid (C18:0) being the best substrate and forming the greatest amounts of alkene product for both mutants (Table 3). Crystal structure data for the H85Q and F79A mutants reveal only minor alterations to OleT_JE_ active site structure, suggesting that quite subtle changes in the positions of active site amino acids, water molecule(s), and the substrate carboxylate region may be sufficient to trigger catalysis when H_2_O_2_ binds to initiate compound 0 formation. Makris's group have revealed how this could lead to substrate hydroxylation by the radical rebound mechanism, involving hydrogen atom abstraction from the substrate by compound I and subsequent “rebound” of the hydroxyl group to the substrate radical (5). However, through their identification and characterization of the transient ferryl-hydroxo compound II, a novel mechanism for substrate decarboxylation was proposed. In this model, compound II abstracts a further electron from the substrate radical to form a diradical or carbocation intermediate (Fig. 1), which decarboxylates to form CO_2_ and the *n* − 1 terminal alkene, with protonation of the ferric-hydroxo species restoring the water-coordinated resting form of the heme iron (15, 39). Further protein engineering studies on OleT_JE_ are now clearly needed to improve its stability and catalytic performance as an alkene producing enzyme and to facilitate its biotechnological application.

## Experimental Procedures

### 

#### 

##### Expression and Purification of OleT_JE_ and OleT_JE_ Mutants

The gene encoding OleT_JE_ from *Jeotgalicoccus* sp. ATCC 8456 was codon-optimized for expression in *E. coli* and cloned into a modified pET15b (GenScript, Cherwell, UK), incorporating a TEV cleavage site with an N-terminal polyhistidine tag. Rationally designed mutants (H85Q, F79A, F79W, F79Y, R245L, and R245E) were created by site-directed mutagenesis, using a QuikChange Lightning kit (Agilent Technologies LDA UK Ltd., Cheadle, UK). The oligonucleotide primers used were: H85Q forward, 5′-ttttaccatcaaccgtctg gattgcgcctttaccaaac-3′; H85Q reverse, 5′-gtttggtaaaggcgcaatccagacggttgatggtaaaa-3′; F79A forward, 5′-gtatggattgcgcctttaccagccagggtattcacaatacgttt-3′; F79A reverse, 5′-aaacgtattgtgaataccctggctggtaaaggcgcaatccatac-3′; F79W forward, 5′-attgcgcctttaccc cacagggtattcacaatacgtttcggc-3′; F79W reverse, 5′-gccgaaacgtattgtgaataccctgtggggtaaaggcgcatt-3′; F79Y forward, 5′ tgcgcctttaccatacagggtattcacaatacgtttcg-3′; F79Y reverse, 5′-cgaaacgtattgtgaataccctgtatggtaaaggcgca-3′; R245L forward, 5′-gatgaacacgttcctgccgctgattgcgatc-3′; R245L reverse, 5′-gatcgcaatcagcggcaggaacgtgttcatc-3′; R245E forward, 5′-ttgatctgatgaacacgttcgagccgctgattgcgatcaatcg-3′ and R245E reverse, 5′-cgattgatcgcaatcagcggctcgaacgtgttcatcagatcaa-3′. *E. coli* strain C41 (DE3) (Lucigen, Middleton, UK) was used as the expression host for WT OleT_JE_ and all mutants. Cells transformed with the plasmids were grown at 37 °C with shaking at 200 rpm in total volumes of 500 ml of 2YT broth containing 50 μg/ml ampicillin. Expression of WT/mutant OleT_JE_ genes was induced with 100 μm isopropyl 1-thio-β-d-galactopyranoside when an OD_600_ of 0.5 was reached. At point of induction, 500 μm δ-aminolevulinic acid was added to all mutants, and 4 μg/ml hemin was also added to the R245L and R245E expression cultures to aid heme incorporation. At this point, the incubation temperature was lowered to 25 °C, and the cells were grown for a further 16 h. The cells were harvested by centrifugation at 6000 rpm at 4 °C using a JLA-8.1000 rotor in an Avanti J-26 XP centrifuge. The cells were resuspended in 100 mm potassium phosphate (pH 8.0), combined, and centrifuged as before. The cell pellet was then frozen at −80 °C until required for purification.

Cells were thawed and resuspended in 200 g/liter extraction buffer (100 mm potassium phosphate, 1 m NaCl, 10% glycerol, pH 8.0) along with SigmaFAST protease inhibitor mixture tablets, EDTA-Free (Sigma-Aldrich) using 1 tablet/100 ml, and 100 μg/ml DNase I (bovine pancreas; Sigma-Aldrich). The cells were lysed using a cell disruptor (Constant Cell Disruption Systems, Daventry, UK) at a pressure of 15,000 p.s.i. The homogenate was then centrifuged at 20,000 rpm at 4 °C for 90 min using a JA-25.50 rotor (Beckman-Coulter Ltd., High Wycombe, UK). The supernatant was removed and incubated overnight at 4 °C with (10 ml/100 g of cell pellet) Ni-IDA chromatographic medium (Generon, Maidenhead, UK). The mixture was then transferred to a column, and the resin bed was washed with 20 column volumes of buffer A (100 mm KP_i_, 750 mm NaCl, 10% glycerol, pH 8.0) containing 50 mm imidazole. The resin was then washed with 2 column volumes of buffer A with 150 mm imidazole, and the protein was eluted in buffer A with 175 mm imidazole. For OleT_JE_ samples not destined for protein crystallization, the protein was extensively dialyzed into buffer A to remove any imidazole and bound fatty acids retained from expression in *E. coli* and then concentrated by ultrafiltration in a Vivaspin (30,000 molecular weight cutoff; GE Healthcare) and transferred to buffer B (100 mm KP_i_, 750 mm NaCl, 20% glycerol, pH 8.0) using a PD 10 desalting column (GE Healthcare). The protein was then snap frozen in liquid nitrogen and stored at −80 °C until required. Protein destined for crystallography was not frozen at this point, and instead TEV protease (∼500 units) was added to the eluted protein to remove the His tag, and the sample was incubated at 4 °C overnight. The S219V variant of TEV protease was expressed using plasmid pRK793 (Addgene plasmid no. 8827) (37). The protein was then exchanged into buffer A using a PD10 desalting column and added to a bed of Ni-IDA resin. Cleaved protein was eluted with buffer A including 20 mm imidazole. This was then dialyzed extensively to remove imidazole and bound lipids. The protein was concentrated using a Vivaspin and then gel-filtered using a Superdex 200 16/600 (GE Healthcare) column pre-equilibrated with buffer C (100 mm KP_i_, 750 mm NaCl, pH 8.0).

##### UV-visible Spectroscopy of WT and Mutant OleT_JE_ Hemoproteins

The UV-visible spectra of WT and mutant forms OleT_JE_ were collected using a Cary 60 UV-visible spectrophotometer (Agilent). Spectra were recorded at ambient temperature for the proteins in buffer A, typically at concentrations of ∼4–6 μm. The ferrous and Fe^II^-CO forms of the hemoproteins were produced under anaerobic conditions using degassed buffer A. Reduced enzymes were formed by the addition of a few grains of solid sodium dithionite to ferric enzymes. The Fe^II^-CO complexes were then formed by slowly bubbling CO gas into reduced OleT_JE_ proteins until no further spectral shift was observed.

##### Fatty Acid Binding Titrations with OleT_JE_ Mutants

Binding of saturated fatty acids (C12:0, C14:0, C16:0, C18:0, and C20:0) to the OleT_JE_ mutants was performed at 25 °C in buffer A. Substrates were dissolved in 100% ethanol, and additions of 0.5-μl aliquots of substrate stocks (of concentration from 1–100 mm, according to the particular substrate and its affinity for the P450s) were made until no further spectral shift was observed. Spectra (800–300 nm) were recorded using a Cary 60 UV-visible spectrophotometer. Difference spectra were obtained by subtracting the ligand-free spectrum of the P450 from the ligand-bound spectra at each substrate concentration. The absorbance difference maximum (Δ*A*_peak_) and minimum (Δ*A*_trough_) were then identified from the individual data sets, and the maximal absorbance change (Δ*A*_max_) was calculated by subtracting Δ*A*_trough_ from Δ*A*_peak_ in each case, using the same wavelength pair throughout each individual titration. Δ*A*_max_ was then plotted against substrate concentration, and the data were fitted using either a hyperbolic (Michaelis-Menten or Morrison equations) (32) or a sigmoidal (Hill) function to determine the dissociation constants (*K_d_* values) for fatty acid substrate binding.

##### Stopped Flow Kinetic Analysis of Substrate Oxidation by OleT_JE_ WT and Mutant Enzymes

An Applied Photophysics SX18 MR stopped flow spectrophotometer with a photodiode array detector (Leatherhead, UK) was used to collect stopped flow absorption data. For the WT, H85Q, F79A, and F79W OleT_JE_ mutants, a 2.5 molar excess of arachidic acid (C20:0) was mixed with these P450 samples (giving a final P450 concentration of 5 μm), and the mixture was passed through a 0.2-μm syringe filter (Sartorius UK Ltd., Epsom, UK) to remove excess lipids from the P450 protein solution. A nearly saturated aqueous solution of lauric acid (C12:0, 250 μm) was mixed with the F79Y mutant (again giving a final P450 concentration of 5 μm) and passed through a 0.2-μm syringe filter as above. The substrate-bound OleT_JE_ proteins were then mixed with different concentrations of H_2_O_2_ (final concentration post-mixing, 6.25–200 μm) in the stopped flow instrument at 25 °C.

Conversion of the fatty acid-bound, HS ferric forms of the OleT_JE_ enzymes to their LS forms was monitored at 421 nm for the OleT_JE_ WT and OleT_JE_ H85Q mutant enzymes and at 423 nm for the OleT_JE_ F79A enzyme. The data were fitted using a single exponential function and using the Pro-Data SX software suite (Applied Photophysics). The observed reaction rate constants (*k*_obs_ values) were plotted against the relevant H_2_O_2_ concentration, and the data were fitted using a hyperbolic (Michaelis-Menten) function (for the WT, F79Y, and H85Q OleT_JE_ enzymes) or using a linear function in the case of the other OleT_JE_ mutants.

##### EPR Analysis of OleT_JE_ Mutants

Continuous wave X-band EPR spectra for the OleT_JE_ WT and mutant P450s were obtained at 10 K using a Bruker ELEXSYS E500 EPR spectrometer with an ER 4122SHQ Super High Q cavity. Temperature was controlled with an ESR900 cryostat (Oxford Instruments, Abingdon, UK). EPR spectra were collected for WT and OleT_JE_ mutants at concentrations of 100–200 μm for ligand-free, arachidic acid-bound, and imidazole-bound forms. Arachidic acid was added to dilute OleT_JE_ protein until the UV-visible spectrum showed no further optical change toward high spin. The enzyme was then concentrated to an appropriate concentration (200 μm for WT, H85Q, and F79A OleT_JE_; 150 μm for R245L OleT_JE_; and 100 μm for the F79W, F79Y, and R245E mutants) (the latter in its substrate-free form), taking into consideration that some of the mutants are prone to precipitation at high concentrations) using a Vivaspin (30,000 molecular weight cutoff).

##### Crystallography of the OleT_JE_ H85Q and OleT_JE_ F79A Mutants

Crystallization trials for the OleT_JE_ H85Q and OleT_JE_ F79A mutant enzymes were carried out with 400 nl (200 nl of protein and 200 nl of precipitant) sitting drops in MRC 96-well plates (Molecular Dimensions, Newmarket, UK). Molecular Dimensions screens Clear Strategy Screen I, Clear Strategy Screen II, PACT premier, JCSG-plus, and Morpheus were used for OleT_JE_ H85Q. Diffracting crystals were taken from well H10 of JCSG-plus (0.2 m ammonium acetate, 0.1 m Bis-Tris, 25% (w/v) PEG 3350, pH 5.5). For OleT_JE_ F79A, C3 Shotgun Screen (SG1) and JCSG-plus screens were used. Crystals formed were used to make a seed stock, and further plates were set up using an optimized screen. Diffracting crystals were grown in 0.2 m MgCl_2_, 0.1 m HEPES, 20% PEG 6000 (pH 7.5). Plates were set up using a Mosquito nanoliter pipetting robot (TTP Labtech, Melbourn, UK). For OleT_JE_ H85Q, ligand-free and arachidic acid-bound proteins were used for crystal trials at a concentration of 11.5 mg/ml. For the OleT_JE_ F79A mutant, arachidic acid-bound enzyme was used at a protein concentration of 13.5 mg/ml. Arachidic acid was bound to the proteins in the same way as described for the EPR studies.

##### Fatty Acid Substrate Conversion Reactions with WT and Mutant OleT_JE_ Enzymes

Fatty acid substrate conversion reactions were set up using 1 μm of wild-type, H85Q, F79A, F79W, F79Y, R245L, and R245E OleT_JE_ enzymes. The proteins were incubated with 200 μm fatty acid (capric acid, lauric acid, myristic acid, palmitic acid, stearic acid, or arachidic acid), and the reactions were started by the addition of 400 μm H_2_O_2_ in a total volume of 500 μl of buffer A. The reactions were incubated at 32 °C with shaking for 30 min. Thereafter, reactions were stopped by acidifying with 20 μl of 37% HCl. For the C10:0, C12:0, and C16:0 reactions, the internal standards 1-undecene, myristic acid (C14:0), α-OH myristic acid, and β-OH myristic acid were added. For the C14:0, C18:0, and C20:0 reactions, internal standards 1-pentadecene, palmitic acid (C16:0), β-OH palmitic acid, and α-OH palmitic acid were added. After completion of the reactions, the mixtures were extracted with an equal volume of dichloromethane. The dichloromethane extract was then dried with anhydrous magnesium sulfate before the product sample was subjected to GC/MS analysis.

##### Characterization of Fatty Acid Oxidation Products Using GC/MS

Analysis of products formed by fatty acid oxidation using WT and mutant OleT_JE_ enzymes was done using an Agilent 6977A series GC/MSD installed with an Agilent HP-5MS UI 30 m × 0.250 mm × 0.25 μm column (helium flow rate, 1.2 ml/min) (Agilent). A 1-μl sample was injected via sandwich injection with 1 μl of *N*,*O-bis*(trimethylsilyl)trifluoroacetamide containing 1% trimethylchlorosilane to derivatize fatty acids and hydroxylated fatty acids. The split ratio was 10:1, and the inlet was set to a temperature of 280 °C. The oven temperature was held at 40 °C for 1 min and then ramped at 10 °C/min to 290 °C, where it was held for 1 min. Electronic ionization was used, scanning ions in the range of 40–550 *m*/*z* at a temperature of 250 °C. For quantitation, external standard calibrations were generated, and the selected ion monitoring mode was used to identify appropriate *m*/*z* ion fragments for substrate and product analysis.

## Author Contributions

A. W. M. and D. L. conceived and coordinated the study. S. M., D. L., and A. W. M. wrote the paper. S. M., J. D. B., and K. L. T. prepared expression constructs, purified enzymes, and performed substrate binding experiments. S. M., H. M. G., K. J. M., A. W. M., and S. E. J. R. collected and analyzed EPR data. S. M., K. J. M., and R. T. B. collected and analyzed GC-MS data from fatty acid oxidation experiments. S. M. crystallized P450 mutants, and C. W. L. and D.L. collected and analyzed crystallographic data. S. M. collected transient kinetic data. All authors reviewed the results and approved the final version of the manuscript.

## Footnotes

2 The abbreviations used are:

Ni-IDA: nickel-iminodiacetic acid
LS: low spin
HS: high spin
BBSRC: Biotechnology and Biological Sciences Research Council.
